# Clinical parameters for predicting radiation-induced liver disease after intrahepatic reirradiation for hepatocellular carcinoma

**DOI:** 10.1186/s13014-016-0663-1

**Published:** 2016-07-02

**Authors:** Yaoru Huang, Shang-Wen Chen, Ching-Chao Fan, Lai-Lei Ting, Chia-Chun Kuo, Jeng-Fong Chiou

**Affiliations:** Department of Radiation Oncology, Taipei Medical University Hospital, 252, Wu Hsing St., Taipei, 110 Taiwan; Department of Radiation Oncology, China Medical University Hospital, Taichung, Taiwan; Department of Radiology, School of Medicine, College of Medicine, Taipei Medical University, Taipei, Taiwan; Department of Radiation Oncology, Saint Mary’s Hospital, Luodong, Taiwan

**Keywords:** Hepatocellular carcinoma, Irradiation, Radiation-induced liver disease, Prognostic factor

## Abstract

**Background:**

Few data are available on the tolerance of reirradiation in patients with hepatocellular carcinoma (HCC). This study determined the clinical parameters contributing to the development of radiation-induced liver disease (RILD).

**Methods:**

We included 36 patients with HCC who received 2 courses of radiotherapy (RT) to the liver. Using α/β = 15 for tumor and α/β =8 for normal liver tissue for biologically equivalent doses in 2 Gy fractions, mean cumulative to the hepatic tumor and normal liver were 87.7 Gy_15_ and 31.1 Gy_8_, respectively. Hepatic toxicities were classified according to the Common Terminology Criteria for Adverse Events, Version 4.0. Clinical data, including liver function test results, radiological study findings, and RT parameters before and after both courses of RT were retrieved for analysis. Using multivariate analysis, logistic regression was used to identify the predictors of RILD, and Cox regression was performed to explore the prognostic factors for overall survival (OS).

**Results:**

Thirteen patients (36 %) developed RILD after reirradiation. Nine of them died because of progressive liver failure without evidence of tumor progression and were categorized to have lethal RILD. A pretreatment Child–Turcotte–Pugh (CTP) score ≥6 was the only predictor of RILD [odds ratio (OR): 15.83, *p* = 0.001] and lethal RILD [OR: 72.56, *p* = 0.005]. In addition, a CTP score ≥6 and the presence of portal vein tumor thrombosis before reirradiation were 2 prognostic factors for OS.

**Conclusion:**

Despite a limited sample size, residual liver function using a preirradiation CTP score ≥6 is a clinical parameter associated with an increased risk of RILD in patients requiring hepatic reirradiation.

## Introduction

Radiotherapy (RT) has become an increasingly employed modality for treating hepatocellular carcinoma (HCC). However, few data are available on the tolerance of the liver to a second course of hepatic RT. The liver is susceptible to radiation damage; therefore, classic or nonclassic radiation-induced liver disease (RILD) has been reported within weeks or months after the administration of RT [1–3]. However, the guidelines for avoiding RILD apply only to patients planning to receive the first course of hepatic RT. Whether the same guidelines are applicable to previously treated patients is unclear. In particular, HCC is commonly accompanied by liver cirrhosis [4–6]. Therefore, investigating hepatic tolerance when a second course of RT is required is critical.

Studies examining clinical parameters for ensuring the safety of reirradiation for recurrent hepatoma are currently lacking. Hence, to optimize patient selection, we conducted this study to identify risk factors associated with hepatic toxicities and treatment outcomes after reirradiation. In this study, hepatic toxicities were classified according to all RILD and lethal RILD events. Our findings can provide information about the safety and selection criteria for patients with HCC requiring hepatic reirradiation.

## Methods

### Patient population

In this study, we retrospectively analyzed the clinical data of patients who received 2 courses of RT to the liver between July 2004 and March 2014 at two hospitals. The inclusion criteria were the following: (1) a diagnosis of HCC based on tissue proof or the typical imaging appearance according to the criteria of the European Association for the Study of the Liver [7]; (2) administration of prior RT for intrahepatic HCC; (3) residual or recurrent hepatic tumors either within or outside the previous irradiation field; (4) residual or recurrent tumors being unamenable to other treatments such as surgery, transarterial chemoembolization, and radiofrequency ablation; (5) an Eastern Cooperative Oncology Group performance score of 0–1; and (6) a Child-Pugh classification of A or B. The evaluation and treatment policies for the included patients were reviewed by a multidisciplinary team, and reirradiation was recommended.

Comprehensive clinical data of all patients were analyzed, including the age at reirradiation, the interval between the 2 courses of RT, liver function test (LFT) results, imaging study findings, and RT parameters before and after both courses of RT. The RT parameters comprised the clinical target volume (CTV), prescribed dose, normal liver reserve volume, and mean liver dose. The LFT results were the hepatitis B virus (HBV) or hepatitis C virus infection status; levels of serum alanine aminotransferase (ALT), serum aspartate aminotransferase (AST), albumin, total bilirubin, and alkaline phosphatase; prothrombin time; and Child–Turcotte–Pugh (CTP) score. In total, 36 patients were included in this study; patient characteristics are listed in Table 1. The median age was 66 years, with a range of 32–80 years.

**Table 1:** Characteristics of all patients

	Patients with RILD	Patients without RILD
Variables	*N* = 13	*N* = 23
Gender		
Male	11	19
Female	2	4
Median age at reirradiation	64	71
	(range, 32 to 77)	(range, 33 to 89)
TNM stage (AJCC 7th edition)		
T1N0M0	2	4
T2N0M0	1	7
T3N0M0	5	9
T4N1M0	1	0
T3N0M1	4	3
Previous local treatment		
Surgery	1	2
TAE/TACE	7	12
RFA/Cryo	2	5
Category of previous hepatitis		
Hepatitis B infection	6	10
Hepatitis C infection	2	6
non-HBV and non-HCV induced	5	7
CTP scores before the 2nd RT	6.3 ± 1.1	5.2 ± 0.5
Interval between 2 courses (months) abutting score for two PTV	8.1 ± 8.1	12.6 ± 12.2
0	4	5
1	2	9
2	7	9
1st RT parameters		
Mean prescribed dose (EQD_2_, Gy)	49.5 ± 8.2	52.5 ± 5.7
Mean CTV (cm3)	392.9 ± 407.0	345.2 ± 565.0
Mean PTV (cm3)	257.9 ± 207.8	314.4 ± 480.7
Mean normal liver dose (EQD_2_, Gy)	20.8 ± 11.3	20.1 ± 8.2
Mean normal liver volume (cm3)	1255.5 ± 569.4	926.5 ± 242.4
2nd RT parameters		
Mean prescribed dose (EQD_2_, Gy)	32.9 ± 14.5	40.6 ± 12.8
Mean CTV (cm3)	139.1 ± 150.2	211.3 ± 343.1
Mean PTV (cm3)	257.9 ± 207.8	314.4 ± 480.7
Mean normal liver dose (EQD_2_, Gy)	12.3 ± 6.6	10.2 ± 5.5
Mean normal liver volume (cm3)	1173.4 ± 622.2	992.5 ± 196.1
Mean cumulative prescribed dose (EQD_2_, Gy)	80.9 ± 17.8	94.4 ± 13.9
Mean cumulative normal liver dose (EQD_2_, Gy)	32.5 ± 15.1	30.5 ± 9.6

*Abbreviation: RILD* radiation-induced liver disease, TNM stage was evaluated before reirradiation; *TAE* transarterial embolization, *TACE* transarterial chemo-embolization, *RFA* radiofrequency ablation, *Cryo* cryotherapy, *RT* radiotherapy, *CTV* clinical tumor volume, *PTV* planning target volume, *EQD2* biologically equivalent doses calculated in 2Gy

Note: abutting score: if reirradiation applied to a PTV separated from the previous PTV because the tumors were located at different segments, this situation was defined as out-field reirradiation (score = 0). For patients receiving reirradiation at the same or adjacent segment, the 2nd PTVs might be partially included in the previous PTV (score 1), or completely included (score 2)

### Target delineation and RT planning

All patients underwent triphasic computed tomography (CT) or magnetic resonance imaging before treatment. The patients were immobilized using a vacuum bag with motion control using abdominal compression. CT simulation was performed using a 3-mm slice thickness with contrast enhancement; the simulation range included the entire liver, both lungs, and both kidneys. The gross tumor volume (GTV) was contoured as visible tumors on CT. The CTV was defined by leaving a 3–5-mm margin in three dimensions from the GTV or from the area adjacent to visible tumors where microscopic tumors were suspected. The planning target volume (PTV) was delineated as a 5-mm margin added to the CTV in three dimensions. The normal liver reserve was defined as the entire liver excluding all PTVs. According to recommended standards, the normal liver reserve was >700 mL, and the mean dose was restricted to ≤23 Gy_2_ [8, 9]. In addition, the median dose to any kidney was less than 20 Gy_2_, the volume of the duodenum or stomach receiving more than 50 Gy_2_ was restricted to <1 mL, the mean dose to the esophagus was less than 34Gy_2,_ and the cumulative doses to the spinal cord from two courses of RT was less than 60Gy_2_ [10]. The same technique and similar constraints were used for both courses of RT.

All patients were treated using intensity-modulated RT (IMRT) with or without image guidance or image-guided tomotherapy. For patients treated with IMRT, static and coplanar procedures were used, whereas the X-ray energy depended on the tumor location. In case of image-guided IMRT, tumor location was verified with daily cone beam CT with or without the implantation of fiducial markers. A dose–volume histogram (DVH) was generated from a computerized planning system for rigorous evaluation. The prescribed doses for the 2 courses of RT in 36 patients were 30–60 Gy and 10–60 Gy with a fraction size of 1.8–3 Gy; 2 of the 36 patients received stereotactic body radiotherapy with a prescribed dose of 35 Gy in 5 fractions.

#### Follow-up and toxicity definition

A weekly clinical visit and routine LFT were rigorously conducted during and after the RT course. After the reirradiation course, these patients underwent scheduled LFTs and clinical assessment at least once in 2 weeks in the initial 3 months and monthly follow-up thereafter. For any patient developing ≥ grade 3 hepatic toxicities or rapid deterioration of liver function resulting in coagulopathy or encephalopathy, RT was discontinued immediately. When the LFT values deteriorated, the patients were admitted for daily evaluation and supportive care. All toxicities were classified according to the Common Terminology Criteria for Adverse Events (CTCAE), Version 4 [11]. Classic RILD was defined as anicteric hepatomegaly and ascites with more than double the upper limit of the normal level of alkaline phosphate [6]. Moreover, according to the CTCAE criteria, nonclassic RILD was defined as ≥ grade 3 hepatic toxicities, with more than 3 times the upper limit of the normal level of blood bilirubin or more than 5 times the upper limit of the normal levels of ALT or AST [12]. Lethal RILD was defined as death directly caused by RT-related progressive hepatic failure before or without evidence of tumor progression.

In addition, we examined the impact of location of the two PTVs on the effect of RILD. Irradiation applied to recurrent tumors situated inside the previous PTV was defined as in-field reirradiation. Conversely, irradiation applied to a PTV separated from the previous PTV because the tumors were located at different segments was defined as out-field reirradiation (*N* = 9). For patients receiving reirradiation at the same or adjacent segment (*N* = 27), we proposed an abutting score for classify the location of two PTVs when assessing the impact of volume effect, as described in Table 1.

### Statistical analyses

The primary analysis was identifying the clinical parameters that could predict RILD and lethal RILD. The secondary analysis was identifying prognostic factors associated with survival after reirradiation. Logistic regression analysis was used to identify the predictors of RILD. Variables with a statistical significance in the univariate analysis were included in the multivariate analysis. Overall survival (OS) was defined as the time from the commencement of the second course of RT to the date of death from any cause or the final follow-up. Survival curves were calculated using the Kaplan–Meier method and log-rank test. Cox regression was performed to explore the prognostic factors for OS. The variables analyzed included age, the pretreatment LFT values (CTP score, albumin, total bilirubin, ascites), PTV, normal liver dose of the reirradiation, cumulative normal liver dose, duration, location of the two PTVs, and the presence of portal vein tumor thrombosis (PVTT). A 2-sided *p* value of <0.01 was considered statistically significant. All statistical analyses were performed using SPSS 13.0 for Windows (SPSS Inc., Chicago, IL, USA).

## Results

### General outcomes

The mean follow-up duration after reirradiation was 17 months (range, 3 to 74 months). Thirteen patients (36 %) developed RILD within 3 months after the initiation of reirradiation. All events were categorized as nonclassic RILD according to their clinical manifestation. Of the 13 patients, 2 could not complete the allocated RT regimen because of hepatic toxicities, and the prescribed doses were 20 Gy in 10 fractions and 21.6 Gy in 12 fractions. There was no evidence to show the continuous deterioration of liver function after the 1st course of RT. Although the previous RT might lead to a subclinical change of liver function, the RILD was mainly attributed to the consequence of reirradiation.

The details of both courses of RT are summarized in the Appendix. We normalized the RT dose by using biologically equivalent doses calculated in 2 Gy as the α/β ratio of 15 for HCC [13] and 8 for the adjacent hepatic tissues [10]. In brief, the mean irradiation doses of the second course of RT for the hepatic tumor and normal liver tissue were 37.52 Gy_15_ (range, 10–58.68 Gy_15_) and 10.83 Gy_8_ (range, 2.5–23.97 Gy_8_), respectively. Moreover, the mean cumulative doses to the hepatic tumor and normal liver tissue were 87.70 Gy_15_ (range, 60.47–117.37 Gy_15_) and 31.11 Gy_8_ (range, 9.15–62.84 Gy_8_), respectively. The median interval between the 2 courses of RT was 11.0 months (range, 1–47 months).

Among the 13 patients with RILD, 4 had transient grade 3 hepatic toxicity, and the LFT values eventually returned to the normal range within 1 month after the completion of RT. Nine patients had lethal RILD without evidence of intrahepatic tumor progression; 8 patients died of fulminant hepatic failure within 3 months, and the remaining patient died of progressive liver failure 5 months after reirradiation. Furthermore, 2 patients did not complete the prescribed RT course because of a rapid deterioration of hepatic function during treatment, which LFT showed more than 5 times the upper limit of the normal levels of ALT or AST or more than 3 times the upper limit of the normal level of blood bilirubin. In addition to the 13 patients with RILD, 2 patients died of massive bleeding caused by esophageal and gastric varices within 6 months after reirradiation, which was a fatal complication of portal hypertension and classified as non-RILD related death. The other treatment-related adverse events such as RT-induced gastrointestinal bleeding, angiocholitis, and biliary stricture were not observed in this cohort.

### Risk factors for RILD and lethal RILD

To investigate the risk factors for RILD, clinical data, including the pretreatment LFT values, CTP score, and RT parameters before both courses of RT, were analyzed. As shown in Table 2, several pretreatment LFT values and clinical parameters were associated with RILD in univariate analysis. Multivariate analysis revealed that a pretreatment CTP score ≥6 was a predictor of RILD [odds ratio (OR): 15.83, 95 % confidence interval (CI): 2.95–85.08, *p* = 0.001]. The results of univariate and multivariate analyses for lethal RILD are summarized in Table 3. A preirradiation CTP score ≥6 was the only factor associated with lethal RILD [OR: 72.56, 95 % CI: 3.650–1442.252, *p* = 0.005]. No statistical differences were observed among all dosimetric parameters between patients with and those without RILD. In addition, the abutting score for the two PTVs and the treatment interval did not affect RILD. Furthermore, the pretreatment LFT before the 1st course of RT and normal liver dose or volume of the first RT course did not affect the occurrence of RILD after reirradiation (Appendix).

**Table 2:** Risk factors associated with RILD after reirradiation

Variables	RILD (+)	RILD (−)	Univariate	Multivariate		
			*p* value	*p* value	OR	95 % CI
Total number	13	23				
Liver function before 2nd RT						
Total bilirubin ≧2.0 mg/dL	4	0	0.016			
Albumin ≦3.5 g/dL	8	4	0.007			
Presence of ascites	3	1	0.086			
INR ≧1.71	0	0				
CTP score ≧6	10	4	<0.0001	0.001	15.833	2.947 ~ 85.075
Presence of PVTT	8	5	0.017			
AST/ALT ≧3x of upper normal limit	5	1	0.088			
Hepatitis						
Hepatitis B infection	6	10	0.877			
Hepatitis C infection	2	6	0.458			
RT parameters of 2nd RT						
Mean CTV (cm^3^)	158.8 ± 157.9	204.2 ± 348.7	0.740			
Mean PTV (cm^3^)	257.9 ± 207.8	314.4 ± 480.7	0.260			
Mean normal liver dose (EQD_2_, Gy_8_)	12.0 ± 6.8	10.2 ± 4.8	0.726			
Mean normal liver volume (cm^3^)	1173.4 ± 622.2	992.5 ± 196.1	0.332			
Interval between 2 courses (month)	8.1 ± 8.1	12.6 ± 12.3	0.247			
Mean cumulative normal liver dose (EQD_2_, Gy_8_)	32.0 ± 15.0	30.6 ± 9.7	0.233			
Mean cumulative prescribed dose (EQD_2_, Gy_15_)	82.8 ± 15.9	90.6 ± 15.4	0.065			
Abutting score for two PTV	1.3 ± 0.9	1.1 ± 0.7	0.548			

*Abbreviation: RILD* radiation-induced liver disease, *OR* odds ratio, *CI* confidence interval, *CTP* Child-Turcotte-Pugh, *PVTT* portal vein tumor thrombosis, *AST* aspartate aminotransferase, *ALT* alanine aminotransferase, *CTV* clinical tumor volume, *NS* no significance, *EQD2* biologically equivalent doses calculated in 2Gy

**Table 3:** Risk factors associated with lethal RILD after reirradiation

Variables	Lethal RILD (+)	Lethal RILD (−)	Univariate	Multivariate		
			*p* value	*p* value	OR	95 % CI
Total number	9	27				
Liver function before 2nd RT						
Total bilirubin ≧2.0 mg/dL	4	0	0.002			
Albumin ≦3.5 g/dL	6	6	0.001			
Presence of ascites	3	1	0.014			
INR ≧1.71	0	0				
CTP score ≧6	8	6	<0.001	0.005	72.5554	3.650 ~ 1442.252
Presence of PVTT	7	6	0.003			
AST/ALT ≧3x of upper normal limit	4	2	0.121			
Hepatitis						
Hepatitis B infection	5	11	0.439			
Hepatitis C infection	1	7	0.355			
RT parameters of 2nd RT						
Mean CTV (cm^3^)	195.2 ± 166.9	186.4 ± 331.7	0.806			
Mean PTV (cm^3^)	315.5 ± 224.5	285.7 ± 447.7	0.890			
Mean normal liver dose (EQD_2_, Gy_8_)	9.7 ± 6.8	11.3 ± 5.2	0.019			
Mean normal liver volume (cm^3^)	1305.8 ± 687.6	975.2 ± 210.5	0.078			
Interval between 2 courses (month)	6.0 ± 8.5	12.7 ± 11.4	0.119			
Mean cumulative normal liver dose (EQD_2_, Gy_8_)	27.9 ± 12.5	32.2 ± 11.5	0.221			
Mean cumulative prescribed dose (EQD_2_, Gy_15_)	75.0 ± 12.7	92.1 ± 14.7	0.005			
Abutting score for two PTV	1.4 ± 0.8	1.1 ± 0.8	0.824			

Abbreviation as Table 2

### Prognostic factors for OS

**Fig. 1:**
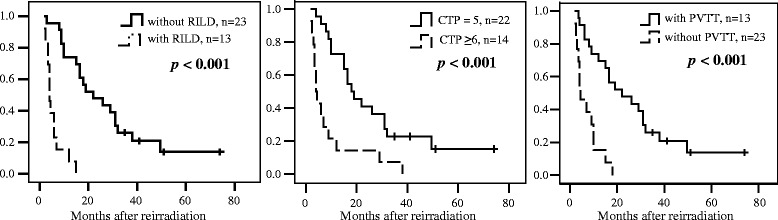
Overall survival according to the occurrence of RILD, pre-reirradiation CTP score, and presence of PVTT. Abbreviation: RILD, radiation-induced liver disease; CTP, Child-Turcotte-Pugh; PVTT, portal vein tumor thrombosis

At the time of this analysis, 32 patients died of disease progression with or without hepatic failure. Patients with RILD exhibited a shorter OS than did those without RILD, with the median survival being 5.7 and 29.0 months, respectively (*p* < 0.001; Fig. 1). The median survival decreased to 3.8 months in patients with lethal RILD. In multivariate analysis, the presence of PVTT [hazard ratio (HR): 12.41, 95 % CI: 2.05–12.41, *p* < 0.001) and a CTP score ≥6 (HR: 8.79, 95 % CI: 1.48–6.74, *p* = 0.003) before reirradiation were prognostic factors. As depicted in Fig. 1, the median OS of patients with and without PVTT was 5 months and 22 months, respectively (*p* < 0.001), whereas that of patients with CTP scores of ≥6 and 5 was 4 months and 18 months (*p* < 0.001), respectively. No statistical difference was observed regarding OS for other clinical parameters, such as age, the irradiation interval, and the dosimetric parameters.

## Discussion

In advanced HCC, RT is assumed to delay the time to tumor progression and resolves vascular damage caused by PVTT. However, the benefits are reduced by the increased risk of RT-related hepatic toxicities. Several studies have reported clinical parameters associated with RILD [14–18]; however, currently, no guidelines exist regarding hepatic tolerance or risk factors for RILD after reirradiation. This study is the first to reveal that a CTP score ≥6 is associated with an increased risk of RILD and predicts the occurrence of lethal RLID. In particular, the estimated OR was more than 10-fold higher than that of patients with a CTP score of 5. In addition, a high mortality rate can be anticipated in patients with a CTP score ≥6. In our study, patients with a pretreatment CTP score ≥6 had a median OS of less than 6 months; therefore, tailored selection is essential for patients requiring a second course of RT.

This study analyzed several pretreatment LFT parameters and concluded that the CTP score, which is widely used for the clinical assessment of liver cirrhosis, was a stronger predictor of hepatic toxicities than the other parameters. In addition, our study revealed that the duration between the 2 courses of RT and the dosimetric parameters did not affect the risk of hepatic toxicities, implying that residual liver function still plays a major role in determining the tolerance to reirradiation. This finding is consistent with the findings of several studies [3, 12, 14–17, 19, 20]. Yoon reported no correlation between RILD and DVH parameters, including the total liver volume, mean liver dose, V30, normal tissue complication probability (NTCP), and equivalent uniform dose [15]. Cheng et al. proposed that patients with HCC having HBV infection and a Child–Pugh class of B have a high risk of RILD [14]. Liang et al. suggested that the severity of hepatic cirrhosis is a major prognostic factor for RILD. In addition, some studies have suggested that the indocyanine green retention rate at 15 min (ICG-R15) is an accurate tool for predicting RILD [15, 16]. Lee et al. indicated that the CTP score is a more reliable predictor of RILD than is the ICG-R15 [21]. By contrast, Pan et al. conducted quantitative analyses of normal tissue in a clinic and suggested applying stricter dose constraints to RT for HCC than for metastatic hepatic tumors, namely mean normal liver doses of less than 28 Gy for HCC and less than 32 Gy for liver metastases for therapeutic partial liver RT (in 2-Gy fractions) [22]. To reach a widely accepted consensus, clinical trials analyzing comprehensive DVH data, clinical parameters, and the HBV titer should be conducted to determine patients with a high risk of RILD after reirradiation.

Although our study showed no impact of location of the two PTVs on RILD, the effect of the dose distribution pattern on outcomes requires further investigation. If recurrent tumors are situated inside the previous PTV, the surrounding hepatic tissues receive a summation of doses from both courses of RT. Dawson et al. proposed that a partial liver can tolerate more than 100 Gy if the volume is smaller than 25 % [5]. By contrast, the cumulative low dose volume is larger when recurrent tumors are far from the previous PVTT. According to a similar NTCP model [5], two-thirds of the total liver volume was assumed to tolerate doses of less than 48 Gy, whereas the total liver volume can tolerate doses of less than 32 Gy. In this study, we compared the cumulative dose to the normal liver rather than the detailed DVH profile because of the difficulty of summing DVHs from both courses of RT. Therefore, comprehensive DVH data should be retrieved for future analysis.

PVTT is an established prognostic factor for survival in patients with HCC receiving hepatic RT [3]. Our study revealed that PVTT had a significant effect on OS after reirradiation. Therefore, in patients with PVTT, the risk of shortened survival should be considered before reirradiation. In a retrospective study of a cohort of 46 patients with HCC receiving RT [3], Furuse et al. reported that PVTT exerted substantial effects on acute adverse hepatic toxicities, survival, and tumor response. They also confirmed that acute hepatic toxicities, including hyperbilirubinemia and hypoalbuminemia, were critical factors affecting survival. Therefore, in the future, we plan to clarify the association between the grade of the tumor thrombus and adverse hepatic events or treatment outcomes.

Our study results should be interpreted with caution. First, the results of this retrospective study with a limited sample size from 2 institutes should be prospectively validated using different irradiation schemes and patient populations. In addition, complete parameters of liver function such as gamma glutamyl transferase or virus titers were imperative to monitor the hepatic damage. Furthermore, the place of SBRT was still unclear in this study because of different biological dose effect compared to conventional radiotherapy. A prospective trial using SBRT for recurrent hepatic tumor is essential to maximize the therapeutic ratio. Finally, the conclusion can be strengthened by collecting and comparing more comprehensive DVH parameters. Thus, a modified NTCP model can be designed for obtaining an accurate score of the risk of RILD following reirradiation. Nonetheless, our study has implications for decision-making regarding initiating the second course of hepatic RT or stratifying patients in clinical trials. With more careful and thorough evaluation, this study can help radiation oncologists more meticulously re-treat patients with HCC, and survival benefits can be expected in patients who do not develop RILD. Early prediction of potential hepatic toxicities would enable administering individualized therapy to patients requiring a second course of hepatic RT.

## Conclusion

Although this study included low number of patients into the analysis, a pretreatment CTP score ≥6 is associated with an increased risk of RILD and lethal RILD in patients requiring a second course of hepatic RT. Patients with RILD have a high mortality rate, implying that residual liver function plays a crucial role in determining the tolerance to reirradiation and survival after treatment. A multicenter prospective study is required to examine additional comprehensive risk factors for RILD.

## Additional information - Ethics approval

This retrospective study was approved by Taipei Medical University - Joint Institutional Review Board with approval no. N201508033.

## Consent for publication

Not applicable.

## Availability of data and materials

Not applicable.

## Abbreviation

ALT, alanine aminotransferase; AST, aspartate aminotransferase; CT, computed tomography; CTCAE, Common Terminology Criteria for Adverse Events; CTP, Child-Turcotte-Pugh; CTV, clinical target volume; DVH, dose–volume histogram; GTV, gross tumor volume; HBV, hepatitis B virus; HCC, hepatocellular carcinoma; ICG-R15, indocyanine green retention rate at 15 min; LFT, liver function test; NTCP, normal tissue complication probability; OS, overall survival; PTV, planning target volume; PVTT, portal vein tumor thrombosis; RILD, radiation-induced liver disease; RT, radiotherapy

## Appendix

**Table 4:** Pretreatment liver function before the 1st course of RT and the association with RILD after reirradiation

Variables	RILD (+)	RILD (−)	Univariate
			*p* value
Total number	13	23	
Liver function before 1st RT			
Total bilirubin ≧2.0 mg/dL	0	0	
Albumin ≦3.5 g/dL	5	3	0.54
Ascites	0	0	
INR ≧1.71	0	0	
CTP score ≧6	4	2	0.77
AST/ALT ≧3x of upper normal limit	3	2	0.38
Presence of PVTT	5	3	0.75
RT parameters			
Mean PTV of the 1st RT (cm^3^)	605.7 ± 551.5	410.6 ± 667.6	0.41
Mean normal liver dose of 1st RT (EQD_2_, Gy_8_)	20.8 ± 12.0	21.3 ± 8.7	0.87
Mean normal liver volume of 1st RT (cm^3^)	1255.5 ± 592.4	1055.3 ± 274.3	0.17

*Abbreviation: RILD* radiation-induced liver disease, *OR* odds ratio, *CI* confidence interval, *CTP* Child-Turcotte-Pugh, *PVTT* portal vein tumor thrombosis, *AST* aspartate aminotransferase, *ALT* alanine aminotransferase, *CTV* clinical tumor volume, *NS* no significance, *EQD2* biologically equivalent doses calculated in 2Gy

## References

[1] Dawson LA, Guha C (2008). Hepatocellular carcinoma: radiation therapy. Cancer J.

[2] Klein J, Dawson LA (2012). Hepatocellular carcinoma radiation therapy: review of evidence and future opportunities. Int J Radiat Oncol Biol Phys.

[3] Furuse J, Ishi H, Nagase M (2005). Adverse hepatic events caused by radiotherapy for advanced hepatocellualr carcinoma. J Gastroenterol Hepatol.

[4] Dawson LA, Haken R (2005). Partial volume tolerance of the liver to radiation. Semin Radiat Oncol.

[5] Dawson LA, Normolle D, Balter J (2002). Analysis of radiation-induced liver disease using the Lyman NTCP models. Int J Radiat Oncol Biol Phys.

[6] Guha C, Kavanagh BD (2011). Hepatic radiation toxicity: avoidance and amelioration. Semin Radiat Oncol.

[7] Son SH, Jang HS, Lee H (2013). Determination of the α/β ration for the normal liver on the basis of radiation-induced hepatic toxicities in patients with hepatocellular carcinoma. Radiat Oncol.

[8] Dawson LA (2011). Overview: Where does radiation therapy fit in the spectrum of liver cancer local-regional therapies?. Semin Radiat Oncol.

[9] Cholongitas E, Papatheodoridis GV, Vangeli M (2005). Systemic review: the model for end-stage liver disease – should it replace child-Pugh’s classification for assessing prognosis in cirrhosis?. Aliment Pharmacol Ther.

[10] Marks LB, Yorke ED, Jackson A (2010). Use of normal tissue complication probability models in the clinic. Int J Radiat Oncol Biol Phys.

[11] Common Terminology Criteria for Adverse Events (CTCAE) v4.0. http://ctep.cancer.gov/protocolDevelopment/electronic_applications/ctc.htm 15818867

[12] Cheng JC, Wu J-K, Huang C-M (2002). Radiation-induced liver disease after radiotherapy for hepatocellular carcinoma: clinical manifestation and dosimetric description. Int J Radiat Oncol Biol Phys.

[13] Hennequin C, Quero L, Rivera S (2011). Radiosensitivity of hepatocellular carcinoma. Cancer Radiother.

[14] Cheng JC, Wu J-K, Lee PC (2004). Biologic susceptibility of hepatocellular carcinoma patients treated with radiotherapy to radiation-induced liver disease. Int J Radiat Oncol Biol Phys.

[15] Yoon HI, Koon WS, Lee IJ (2012). The significance of ICG-R15 in predicting hepatic toxicity in patients receiving radiotherapy for hepatocellular carcinoma. Liver Int.

[16] Stenmark MH, Cao Y, Wang H (2014). Estimating functional liver reserve following hepatic irradiation: adaptive normal tissue response models. Radiother Oncol.

[17] Llovet JM, Ducreux M, Lencioni R (2012). European association for the study of the liver EASL–EORTC clinical practice guidelines: management of hepatocellular carcinoma. J Hepatol.

[18] Que JY, Lin LC, Lin KL (2014). The efficacy of stereotactic body radiation therapy on huge hepatocellular carcinoma unsuitable for other local modalities. Radiat Oncol.

[19] Liang S-X, Zhu X-D, Xu Z-Y (2006). Radiation-induced liver disease in three-dimensional conformal radiation therapy for primary liver carcinoma: the risk factors and hepatic radiation tolerance. Int J Radiat Oncol Biol Phys.

[20] Xu Z-Y, Liang S-X, Zhu J (2006). Prediction of radiation-induced liver disease by Lyman normal tissue complication probability model in three-dimensional conformal radiation therapy for primary liver carcinoma. Int J Radiat Oncol Biol Phys.

[21] Lee IJ, Seong J, Shim SJ (2009). Radiotherapeutic parameters predictive of liver complications induced by liver tumor radiotherapy. Int J Radiat Oncol Biol Phys.

[22] Pan CC, Kavanagh BD, Dawson L (2010). Radiation-associated liver injury. Int J Radiat Oncol Biol Phys.

